# Understanding the neurobehavioural impact of Duchenne muscular dystrophy: A multicentre European study

**DOI:** 10.1007/s00787-026-02988-7

**Published:** 2026-04-28

**Authors:** Anna Kolesnik, Chloe Geagan, Pien Weerkamp, Ruben Miranda, Anna Slipsager, Isabelle Desguerre, Volker Straub, Jos Hendriksen, Eugenio Mercuri, Francesco Muntoni, David Skuse, the BIND Consortium, Nathalie Angeard, Dimitrios Athanasiou, Mariana Suárez Bagnasco,, Suzie-Ann Bakker, Nathalie Boddaert, Daniela Pia Rosaria Chieffo, Ellie Drummond,, Chloe Durrlemann, Luis Miguel García-Moreno, Fernanda Fortunato, Rosanne Govaart, Irina Guliaeva, Catherine Hankinson, Leslie Hemar, Rebecca Hendel, Landon Gregory, Monika Malinova, William Mandy, Federica Moriconi, Erik Niks, Monia Pellizzari, Sarah Poncet, Elvina Sakellariou, Sergiu Siminiuc, Lily Smythe, Pietro Spitali, Mads Peter Godtfeldt Stemmerik, John Vissing, Asmus Vogel, Elizabeth Vroom, Jeanne Wolstencroft

**Affiliations:** 1 Dubowitz Neuromuscular Center, GOS Institute of Child Health, UCL, London, UK; 2 John Walton Muscular Dystrophy Research Centre, Newcastle University and Newcastle Hospitals NHS Foundation Trust, Newcastle, UK; 3 Kempenhaeghe Centre for Neurological Learning Disabilities, Heeze, The Netherlands; 4 School for Mental Health and Neuroscience, Maastricht University, Maastricht, The Netherlands; 5 Department of Psychobiology and Methodology in Behavioural Sciences, Universidad Complutense de Madrid, Madrid, Spain; 6 Copenhagen Neuromuscular Centre, Department of Neurology, Rigshospitalet, Copenhagen, Denmark; 7 Imagine Institute des maladies genetiques Necker Enfant maladies foundation, Paris, France; 8 Department of Paediatric Neurology, Catholic University, Rome, Italy

**Keywords:** Duchenne muscular dystrophy, Neurobehavioural problems, ADHD, Parent-report

## Abstract

Duchenne muscular dystrophy (DMD) is a severe X-linked disorder characterised by dystrophin deficiency, leading to progressive muscle degeneration and reduced life expectancy. Recent studies report neurodevelopmental and psychiatric comorbidities, though their manifestations remain only partially understood. In this multicentre European study, conducted within the Brain Involvement iN Dystrophinopathies (BIND) consortium, we assessed neurobehavioural outcomes in 238 males aged 5–17 years with genetically confirmed DMD, using standardised parent-report questionnaires. Parent-reported symptoms were reviewed and rated by trained clinicians using criteria from the Diagnostic and Statistical Manual of Mental Disorders (DSM-V^1^). We compared findings with two UK-based cohorts: (1) children with pathogenic copy number variants (CNVs) from the IMAGINE-ID study [2] (*n* = 470; matched on sex, chronological age, and parent-estimated mental age), and (2) a male normative population sample [3] (*n* = 3,851). Clinician-rated Development and Well-Being Assessment (DAWBA) assessments indicated that 21% of participants met criteria for at least one DSM-5 diagnosis, including attention-deficit/hyperactivity disorder (ADHD, 8.4%), anxiety-type disorders (7.9%), and autism spectrum disorder (ASD, 6.7%). Genotype-phenotype analyses within DMD revealed elevated ASD prevalence in individuals with Dp140– variants, while ADHD and anxiety were distributed across genotypic groups. Although the DMD cohort exhibited a less severe neurobehavioral impact than the IMAGINE-ID group, they showed markedly elevated emotional and behavioural difficulties as well as prevalence of DAWBA-based diagnoses compared to normative population data. Our findings underscore the neuropsychiatric burden associated with DMD and support the need for routine screening and stratified care approaches informed by genotype.

**Supplementary Information:**

The online version contains supplementary material available at 10.1007/s00787-026-02988-7.

## Introduction

Duchenne muscular dystrophy (DMD) is a severe and progressive neuromuscular disorder that primarily affects males due to its X-linked recessive inheritance pattern. The disorder is caused by mutations in the *DMD* gene, which lead to an absence or deficiency of dystrophin, a protein essential for maintaining muscle integrity. The average life expectancy for individuals with DMD has improved with advances in long-term steroid regimes and supportive care, but remains significantly reduced, limited to third or fourth decade of life.

There is a growing body of research on neurodevelopmental and psychiatric dimensions of the disorder [1–3]. Brain involvement in DMD has long been recognised [4], yet only recently has been investigated systematically. This apparent gap has a significant impact on care delivery due to lack of evidence-based standards, and negative outcomes for patients [5]. A recent workshop argued that improvement in the psychological care of patients affected by DMD is critical, and called for research concerning psychological, neurocognitive and neuropsychiatric needs [6]. We aimed to better identify those needs by means of large multicentre European study. Our findings contribute to a more nuanced understanding of DMD and its management.

## Central nervous system (CNS) involvement in Duchenne muscular dystrophy

Recent studies report that certain pathogenic variants within the DMD gene are associated with an elevated likelihood of neuropsychiatric and neurocognitive problems [1, 3]. Specifically, authors suggest that dystrophin alterations in the CNS contribute to a higher incidence of neurobehavioral and mental health difficulties, including attention-deficit/hyperactivity disorder (ADHD), autism spectrum disorder (ASD), and anxiety disorders. Human neuroimaging studies have shown these behavioural characteristics are associated with CNS involvement in DMD, including both structural and functional brain alterations [7].

**Fig. 1:**
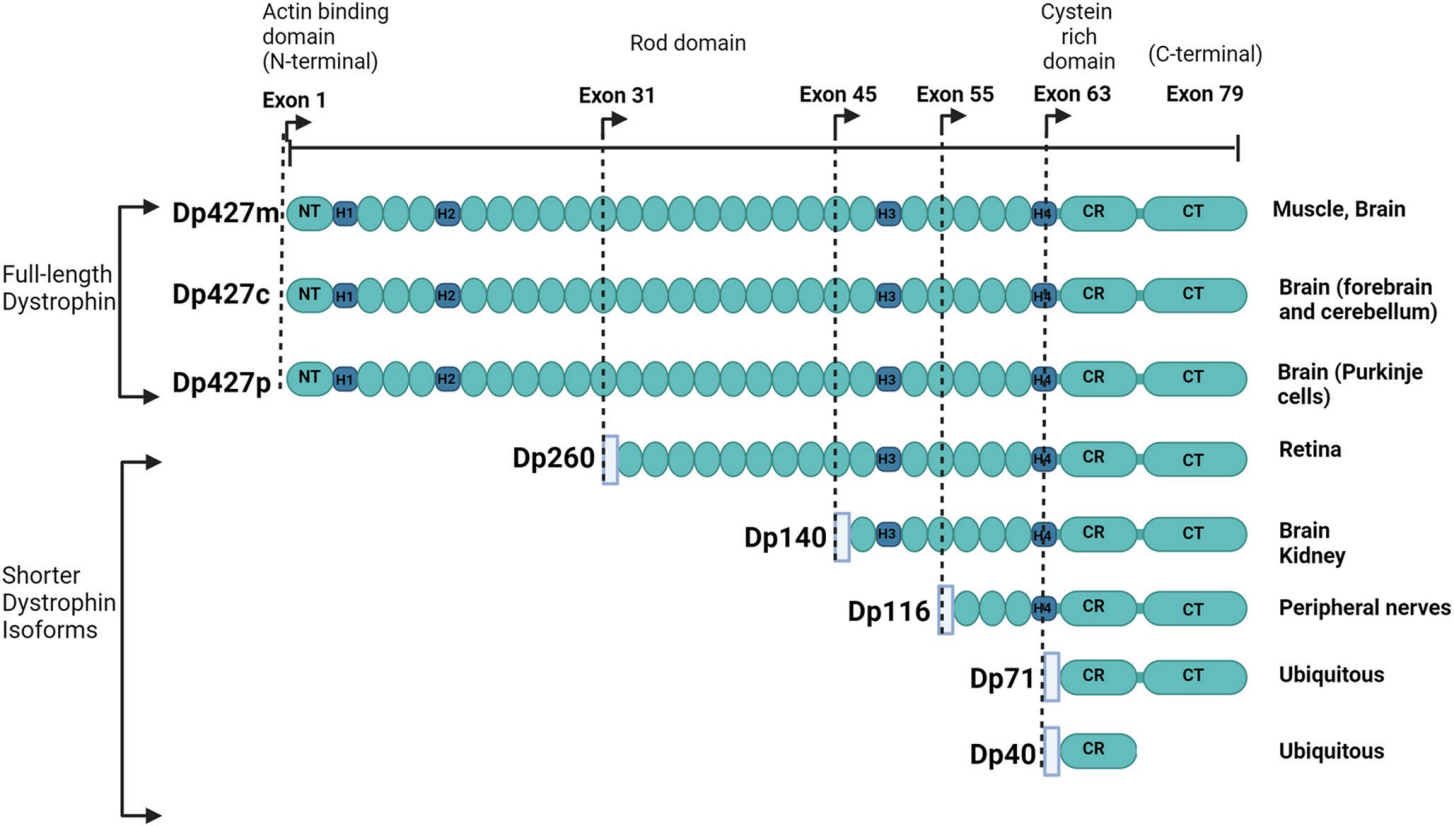
Organisation of the *DMD* gene and its associated dystrophin isoforms, including Dp427c, Dp140 and Dp71 involved in brain function *(with permission from Vaillend et al.*,* 2025)*

On the basis of both human and animal studies, DMD isoform mutations are known to impact neuroplasticity, neurotransmitter signalling, white matter connectivity as well as overall cortical volume [8–10]. Specific dystrophin isoforms, such as Dp427, Dp140, and Dp71, are expressed in the brain (Fig. 1) and play crucial roles in neuronal function and synaptic plasticity [11–14]. Mutations located between exons 1–44 affect the expression of Dp427 in the brain are typically associated with a milder phenotype. Mutations located between exons 51–62 of the gene affect both Dp427 and Dp140 and are associated with moderate levels of impairment. Mutations located at the 3’ end of the gene are found in only ~ 6% of DMD boys (exons 63–79), abolish expression of all isoforms and are associated with the most severe CNS phenotype [13, 14].

The neurocognitive consequences associated with brain involvement in DMD have been consistently reported [15, 16]. On average, children with DMD have intelligence quotient (IQ) scores approximately one standard deviation (SD) below the population mean. Many children experience specific learning difficulties such as dyslexia and deficits in verbal working memory, with a potential link between site of the mutation and degree of cognitive functioning [17].

Alterations in brain dystrophins, including Dp427, have been associated with changes in gamma-aminobutyric acid (GABA) receptor function, which is critical for inhibitory neurotransmission thereby increasing susceptibility to anxiety-type behaviours, as reported in DMD mouse models [14, 18].

### Prevalence of neurobehavioural diagnoses in DMD

The prevalence of neuropsychiatric conditions in DMD has been widely documented, though estimates vary widely across studies. Reported rates of ADHD range from 0 to 50%, ASD from 0 to 21%, anxiety disorders from 7 to 60%, and obsessive-compulsive disorder (OCD) from 5–33% [1, 3, 19]. Depression has been observed in certain cohorts, with prevalence rates varying and often emerging as a secondary consequence in young adults with DMD [1, 20, 21].

These discrepancies in apparent prevalence are largely attributable to methodological differences. Prior studies have employed a range of assessment tools, including structured clinical interviews [3, 22], registry-based data [23, 24], and parent-report questionnaires [19, 25]. The use of different diagnostic frameworks (e.g., the *Diagnostic and Statistical Manual of Mental Disorders* [DSM] and the *International Classification of Diseases* [ICD]), sample sizes, and recruitment settings (specialist clinics vs. population-based samples) further contributes to variability. In some cases, psychiatric symptoms may be under-recognised due to the overshadowing effects of physical disability or limited access to psychological services [6, 14]. Addressing this issue requires studies that capture the full spectrum of neurobehavioural symptoms in large, unselected samples, with adequate comparison groups, to evaluate the interplay between physical disability, genotype and neuropsychiatric presentations. A recent meta-analysis of 23 studies highlighted this heterogeneity, underscoring the need for standardised, large-scale investigations [1]. As a focus for investigating genotype–phenotype associations it has been proposed to study mutations affecting specific dystrophin isoforms (e.g., Dp140, Dp71) and to evaluate potential links to an increased risk of neurodevelopmental impairments [1].

The latest standards of care for DMD emphasise the need for screening for psychosocial and neurodevelopmental conditions [28], but lack specific recommendations for addressing such comorbidities. Addressing this gap requires comprehensive studies and international collaboration, that integrate validated diagnostic tools, genotype data, and appropriate comparison groups in a generalisable way. Such efforts are essential to inform evidence-based care and improve outcomes for individuals with DMD.

### Present study

This study represents a multi-site European collaboration aimed at improving understanding of neurodevelopmental and emotional difficulties in individuals with DMD, conducted within the Brain Involvement iN Dystrophinopathies (BIND) consortium. Consistent clinical assessment protocols and shared measurement tools were used across participating centres to enable robust cross-site comparisons [29].

To contextualise findings, data from the DMD cohort were compared with population norms from the UK National Survey of Children’s Mental Health 2017 [30], and with a UK-based cohort of children with intellectual disability and known genetic diagnoses from the IMAGINE-ID study (Intellectual Disability and Mental Health: Assessing the Genomic Impact on Neurodevelopment) [31]. This comparative framework allows a more nuanced interpretation of prevalence rates and symptom profiles. Given evidence that loss of the brain‑expressed Dp140 isoform is linked to higher neurodevelopmental risk [3, 18], outcomes were compared by Dp140 status to clarify genotype-phenotype associations and inform clinical stratification.

## Objectives

1. To estimate the **prevalence of neurodevelopmental and emotional disorders** in paediatric DMD population using harmonised clinical data from multiple European sites.
2. To examine **genotype-phenotype associations**, with a focus on the role of Dp140 isoform involvement in cognitive and emotional outcomes.
3. To explore **age-related trends **in the presentation of neurodevelopmental and emotional difficulties across childhood and adolescence.

## Hypotheses

- Individuals with DMD will show elevated rates of neurodevelopmental and emotional disorders compared to population norms. Symptom profile is expected to differ between BIND and IMAGINE-ID cohort due to unique neurodevelopmental impacts of each population.
- Genotypic variants affecting Dp140 expression will be associated with increased risk of cognitive and emotional difficulties.
- The presentation of these difficulties will vary with age, reflecting developmental changes and disease progression.

## Methods

### Study design and participants

We present data from 238 males with DMD (age *M* = 10.26, *SD* = 3.4, *range* 5–17), recruited as part of the BIND study (Part 1, 2020–2024; see Supplementary Appendix 1 for details of recruitment sites). Participant information sheets were made available to eligible and interested participants, through clinic visits, email, post, or a link through the BIND website (https://bindproject.eu/). Interested families were contacted within 24 h to discuss participation in the study. Consent was obtained via phone, videoconference, or online link. Participants were recruited via neuromuscular clinics, patient registries, and advocacy groups across Europe.

Participants inclusion criteria were:

(i) Male;
(ii) Aged between 5 and 17 years;
(iii) Has a genetically proven diagnosis of DMD;
(iv) Has a pathogenic variant of: Dp427 only (Group 1: Dp140+); Dp140 isoform status was unknown (Group 2: Dp140Unk); their pathogenic variants included both Dp427 and Dp140 (Group 3: Dp140-); had pathogenic variants of all isoforms (Group 4: Dp71-).

Exclusion criteria included:

(i) Lack of molecular diagnosis of DMD;
(ii) Pathogenic variant falling outside of the regions of interest;
(iii) Presence of a severe co-morbidity, defined as any medical or psychiatric condition requiring hospitalisation, surgical intervention, or intensive treatment within six months of study commencement, which could interfere with participation or compromise well-being.

Two separate analyses were used to contextualise neurobehavioural difficulties in DMD. Firstly, we selected data from IMAGINE-ID study [31], a prospective UK-based cohort assessing mental health in 2,770 children with intellectual disability and relevant pathogenic variants. From this cohort, a matched sample of 470 children and adolescents (male, ages 6–17, *mean age* = 10.35 years, *SD* = 3 years) with pathogenic copy number variants (CNV) was identified. A range of CNVs was present in the IMAGINE-ID study, including deletions and duplications at loci such as 1q21.1, 2p16.3, 9q34.3, 15q11.2, 15q13.3, 16p11.2, and 22q11.2 [32]. No direct comparisons were made between specific CNVs and the DMD group, due to the heterogeneity in clinical presentation and underlying genetic mechanisms across CNVs [33]. The matching procedure was based on chronological age and parent-estimated mental age, aiming for mild-to-moderate intellectual disability, defined as a mental age within two years of chronological age. DAWBA ratings were carried out by the IMAGINE team at UCL using DSM-5 criteria.

The second comparison group was derived from from the normative population of the UK, collected as part of the national survey *‘Mental Health of Children and Young People in England 2017’; MHCYP* [30], using ICD-10 diagnostic criteria. The main outcome measures were assessed in the same way as in the BIND and IMAGINE-ID cohorts. From this cohort, we have summary data for 3,042 males aged 6–16 years old for the Strengths and Difficulties Questionnaire (SDQ) and up to 3,851 males aged 5–19 years old for the DAWBA assessments. Note that due to safeguarding of individual datasets, only averages and confidence intervals (CIs) were available for statistical comparison. Table 2 provides the demographics of the two comparison groups.

**Table 1:** Descriptive variables of the DMD group, DAWBA diagnoses and SDQ scores separated by genotype group. SDQ categories were grouped based on a 4-band categorisation (Supplementary Appendix 2).

Age (M, SD, range)	Dp140+ (*n* = 105)	Dp140Unk (*n* = 47)	Dp140- (*n* = 77)	Dp71- (*n* = 8)**	Sig. testing*
	10.62 (3.2, 5–17)	9.64 (3.2, 5–16)	10.27 (3.6, 5–16)	10.13 (2.9, 6–15)	*F*(3,237)=0.88, *p*=.448
General health*	**(*n*** **= 67)**	**(*n*** **= 34)**	**(*n*** **= 54)**	**(*n*** **= 3)**	*X*^*2*^(6,156)=0.4.48, *p*=.612
Very good	7	6	9	-	
Good	23	7	16	1	
Fair	35	18	25	2	
Bad	2	3	4	-	
**DAWBA**	**(*n*** **= 105)**	**(*n*** **= 47)**	**(*n*** **= 77)**	**(*n*** **= 8)**	
Any disorder (% of total)	22 (21%)	7 (14.9%)	18 (23.4%)	3 (37.5%)	*X*^*2*^(2,230) = 1.35, *p*=.478
ADHD	10 (9.5%) (	2 (4.2%)	8 (10.3%)	0	
ASD	2 (1.9%)	3 (6.4%)	11 (14.3%)	0	
Anxiety-type disorders***	8 (7.6%)	2 (4.2%)	6 (7.8%)	3 (37.5%)	
ODD	5 (4.7%)	0	0	0	
**SDQ total categories**	**(*n*** **= 105)**	**(*n*** **= 47)**	**(*n*** **= 77)**	**(*n*** **= 8)**	*X*^*2*^(6,230) = 9.51, *p*=.147
Close to average (0–13)	73 (69.5%)	36 (76.6%)	46 (59.7%)	4 (50%)	
Slightly raised (14–16)	14 (13.3%)	3 (6.4%)	12 (15.6%)	2 (25%)	
High (17–19)	8 (7.6%)	2 (4.3%)	13 (16.9%)	1 (12.5%)	
Very high (20–40)	10 (9.5%)	6 (12.8%)	6 (7.8%)	1(12.5%)	

*Parent-reported general health status (DAWBA-derived variable). **Dp71- group excluded from between-group comparisons due to small sample size. ***Anxiety-type disorders comprise the following diagnoses: generalised anxiety, social phobia, separation anxiety, specific phobia.

### Materials

Parents completed online versions of the DAWBA and the SDQ. The DAWBA is a package of parent or self-report interviews and questionnaires that provides DSM-5 and ICD-10 compatible psychiatric diagnoses and broader measures of family functioning, including general health and parental wellbeing [34]. The assessment is available in over 20 languages, including all BIND study languages. The DAWBA assessment offers the option of automated likelihood ratings of psychiatric diagnoses. These were not used, due to concern of their accuracy relative to clinician assessments [35]. Researchers from each BIND site received training at the UCL site in September 2022 to learn about the functions of the computerised interview procedure, and to ensure consistency in interpretation.

Finally, after data collection was complete, all diagnostic decisions were reviewed (translated to English if necessary) by expert UCL-based clinicians who had already established inter-rater reliability with the UK’s MHCYP study team. This procedure ensured consistency in the interpretation of information provided by families about their child’s mental health across all participating sites. For the purpose of the present study and based on previous literature of reported problems in DMD, the following 11 DSM-5 defined diagnoses were pre-selected by the BIND consortium: ASD, ADHD, tics, anxiety-type disorders (separation anxiety, social phobia, specific phobia, generalised anxiety), major depression, oppositional defiant disorder (ODD), conduct disorder (CD), OCD [29].

The SDQ is a brief and comprehensive screening tool used in assessment of mental health comorbidities in children and adolescents with chronic disease [36]. The questionnaire contains 25 items, each with 5 scales measuring: emotional symptoms, conduct problems, hyperactivity & inattention, peer relationship problems and prosocial behaviour. It contains a further 8-item impact assessment, looking at chronicity, distress and burden on the family and daily functioning. Higher scores on the SDQ are associated with more reported difficulties, with prosocial behaviour omitted from the overall total. Scores above the 90th percentile (SDQ total ≥ 17) indicate a high probability of meeting clinical criteria for a DSM-5 diagnosis [37–39]. Scoring of the SDQ subscales is based on a four-tier system; close to average, slightly raised/lowered, high/low and very high/very low (Supplementary Appendix 2). Scores were not shared with participating families. If parents expressed concerns about their child’s behaviour, they were advised to follow up with local services.

### Statistical analyses

Four sets of statistical analyses were conducted. First, descriptive statistics were calculated for DAWBA-based diagnoses and SDQ scores. Next, ANOVAs were performed to examine differences in key outcome variables between genotype groups (DAWBA-based diagnoses, SDQ scores), with Bonferroni adjustments applied to control for multiple comparisons. To explore age-related trends in neurodevelopmental and emotional difficulties, participants were grouped into age bands (e.g., 5–8, 9–12, 13–17 years), and group comparisons were conducted using χ² tests for DAWBA and ANOVAs for SDQ scores. Independent *T*-tests and χ² tests were carried out to compare the key outcome variables between BIND and IMAGINE-ID cohorts. Then, summary data (i.e. means, proportions, *CI*s) from the BIND cohort were compared to UK normative data from the MHCYP 2017 survey. Where overall group differences are observed, follow-up analyses were conducted using relevant covariates to account for potential confounding influences. These included age (as a continuous variable) and health status. All analyses were conducted using IBM SPSS Statistics (Version 28).

## Results

Of the 238 DMD participants, genotype grouping was as follows: 105 were classified as Dp140+ (mutations in exons 1–44), 48 as Dp140 unknown (mutations in exons 45–50), 77 as Dp140- (mutations in exons 51–62), and 8 as Dp71- (mutations in exons 63–79; Table 1). There was no significant difference in mean age between all genotypic subgroups (*p*=.448) or between BIND and IMAGINE-ID cohort (*p*=.358). Due to the small size of the Dp71- group (*n* = 8), it was excluded from inferential analyses for genotypic group comparisons in DMD.

### DAWBA-based diagnoses

Among participants within the BIND cohort who had completed DAWBA parent-report interviews, 21% were rated positive for at least one DSM-5 compatible diagnosis (Any Disorder). The most frequently diagnosed conditions were ADHD, present in 8.4% of participants, anxiety-type disorders in 7.9%, and ASD in 6.7% (Table 1). No cases of CD, depression or OCD were recorded in our sample. For analysis of Parental Wellbeing and General Health between children who screened positive or negative for a DAWBA-based diagnosis, see Supplementary Appendix 3.

**Fig. 2:**
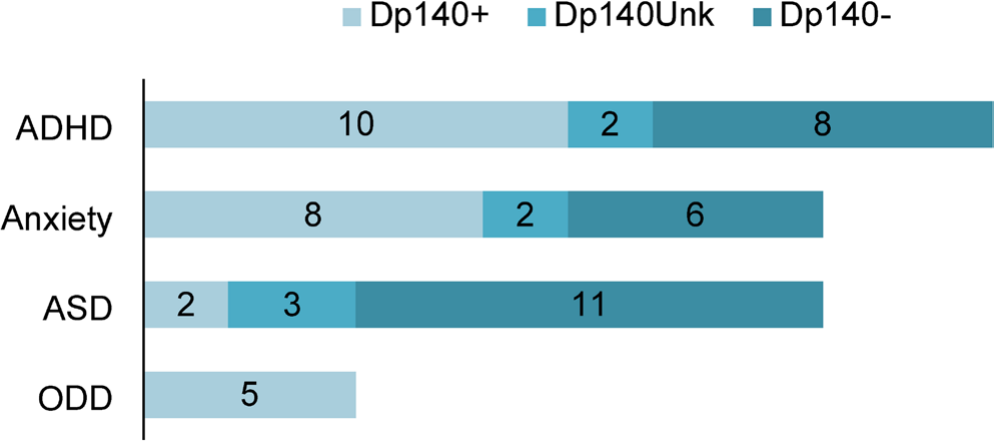
DAWBA-based diagnoses by dystrophin isoform group. Each bar shows the number of individuals meeting criteria for a specific diagnosis within each genotype group

The prevalence of diagnoses varied by dystrophin isoform group, with 21% of individuals in the Dp140 + group, 14.9% in the Dp140Unk group, 23.4% in the Dp140- group meeting criteria for Any Disorder (Fig. 2). Differences in prevalence of Any Disorder across genotype groups were not statistically significant (*X*^*2*^(2,230) = 1.44, *p*=.487). When examining individual diagnoses, however, significant associations were found for ASD (*X*^*2*^(2,230) = 10.57, *p*=.005) and ODD (*X*^*2*^(2,230) = 6.01, *p*=.048). ASD was most prevalent in the Dp140- group, with 11 out of 77 individuals (14.3%) meeting criteria, compared to 5/105 (4.8%) in the Dp140 + group and 1/47 (2.1%) in the Dp140Unk group; (*p*.=005). All five cases of ODD were observed exclusively in the Dp140 + group (*p*=.048; Fig. 2). Notably, a subset of participants (9 individuals, 18% of those who were screened positive for a DAWBA diagnosis) received multiple diagnoses, with ADHD and ASD co-occurring most frequently (Supplementary Appendix 4).

No statistically significant differences were observed in the prevalence of Any Disorder across age groups (5–8 years, 9–12 years, and 13–17 years; *χ²*(2)=0.076, *p*=.963). Similarly, when individual diagnoses (anxiety-type disorders, ASD and ODD) were analysed separately, no significant age-related associations were identified (*p* value ranges between 0.083 and 0.715). While ADHD was more frequently observed in the 5–7 years category (12/20 cases), this trend did not reach significance (*χ²*(2) = 5.93, *p*=.051).

Comparison with the IMAGINE-ID cohort revealed relatively fewer DAWBA-rated clinical diagnoses in participants with DMD relative to the IMAGINE-ID group (DMD 21% compared with 31.5%, *p* =.003; Table 2). ADHD was significantly less prevalent in the DMD cohort (8.4%) compared to IMAGINE-ID (31.1%, *p* <.001), as was ASD (6.7% in DMD vs. 40.6%, *p* <.001). Anxiety-type disorders were also less prevalent in the DMD cohort (7.9%) than in IMAGINE-ID (14.2%, *p* =.016). ODD was rare in the DMD group (2.1%) but significantly more common in the IMAGINE-ID cohort (19.4%, *p* <.001).

Comparison between DMD and normative population from the CMHYP (2017) cohort revealed significant differences (Table 3). The normative population had DAWBA-based diagnoses in 12.6% cases (*Z* = 3.1, *p* <.001). ADHD was significantly more frequent in the DMD cohort (8.4%) compared to 2.62% in CMHYP study, (*Z* = 3.18, *p* <.001), as was ASD (6.72% in DMD vs. 1.92%, *Z* = 2.93, *p* <.001). Anxiety-type disorders were also more prevalent in the DMD cohort (7.9%) compared with 0.8%, (*Z* = 4.07, *p* <.001). ODD was less common in the DMD group (2.1%) compared to the normative population (3.6%), though this difference was not statistically significant (*Z* = −1.53, *p* =.12).

## Strengths and difficulties questionnaire (SDQ)

The mean SDQ total score for the DMD cohort was 10.7 (SD = 6.5), which is slightly above the average score for the normative score for age and sex matched controls (*M* = 8.7; *p*<.001; Table 3 [28]). The distribution of scores indicated that 67.2% of the DMD sample fell within the “close to average” range (scores 0–13), 13% had “slightly raised” scores (14–16), 10% had “high” scores (17–19), and 9.6% fell in the “very high” category (20–40), indicating potential clinical concern. Shapiro-Wilks test for normality showed non-normal distribution of raw scores across all subscales of the SDQ (*p*s<0.003). A logarithmic transformation was applied, to obtain a distribution approximating normality, and results were back-transformed to ease interpretation.

A one-way ANOVA comparing results of genotype groups (Dp140+, Dp140-, Dp140Unk) revealed no significant differences in SDQ Total scores between genotype groups (*F*(3,230)=0.117, *p*=.838). A further multivariate ANOVA conducted on each of the SDQ subscales using logarithmically transformed values did not reveal any significant group differences (all *p* values > 0.15).

SDQ total and subscale scores were compared across three age groups: 5–8 years, 9–12 years, and 13–17 years. A one-way ANOVA revealed no significant effect of age group on SDQ total score (*F*(2,230) = 1.33, *p*=.267). However, significant age-related differences were observed for several subscales: emotional symptoms (*F*(2,230) = 5.92, *p*=.003), conduct problems (*F*(2,230) = 7.9, *p*<.001), and hyperactivity (*F*(2,230) = 7.95, *p*<.001). Bonferroni-corrected post-hoc comparisons revealed that emotional symptoms increased with age, with older participants (13–17 years) reporting significantly higher scores than youngest group (*MeanDiff* = 1.39, *p*=.002). In contrast, conduct problems (*MeanDiff*=−1.07, *p*<.001) and Hyperactivity symptoms (*MeanDiff*=−1.3, *p*=.004) decreased with age.

SDQ total and subscale scores were compared between males with DMD in the BIND cohort and the age and sex-matched comparison group from the IMAGINE-ID study. The mean of the SDQ total score was 21.74 in the IMAGINE-ID cohort, substantially higher than the DMD population (*p*<.001). A series of independent samples t-tests were conducted on log-transformed data. Overall, the IMAGINE-ID cohort displayed significantly higher levels of problems across all SDQ subscales, including greater emotional distress, conduct problems, hyperactivity, peer relationship difficulties, and overall functional impact as well as lower prosocial behaviour relative to the DMD cohort, all *p* values < 0.001 (Table 2).

**Table 2:** Comparison of DAWBA-based diagnoses and SDQ total and subscale scores between BIND cohort, matched comparison group from the IMGAINE-ID cohort

Age (M, SD, range)	DMD Group (BIND) (*n* = 238; *M*,* SD*)	IMAGINE-ID Cohort (*n* = 470; *M*, *SD*)	Sig. testing
	10.7 (6.5, 0–34)	10.35 (3, 6–17)	*t*(706)=−0.363, *p*=.358
**DAWBA**			
Any disorder (% of total)	50 (21%)	148 (31.5%)	χ²(1,708) = 8.62, *p*=.003
ADHD	20 (8.4%)	146 (31.1%)	χ²(1,708) = 45.2, *p*<.001
ASD	16 (6.7%)	191 (40.6%)	χ²(1,708) = 87.84, *p*<.001
Anxiety-type disorders	19 (7.9%)	67 (14.2%)	χ²(1,708) = 5.82, *p*=.016
ODD	5 (2.1%)	91 (19.4%)	χ²(1,708) = 40.16, *p*<.001
**SDQ**			
Total score	10.76 (6.54)	21.74 (6.83)	*t*(706)=−20.94, *p*<.001
Emotional symptoms	2.58 (2.49)	5.24 (2.79)	*t*(532.5)=−12.89, *p*<.001
Conduct problems	1.86 (1.82)	3.78 (2.57)	*t*(631.6)=−11.48, *p*<.001
Hyperactivity problems	3.96 (2.65)	7.87 (2.11)	*t*(397.4)=−19.94, *p*<.001
Peer problems	2.38 (1.98)	4.86 (2.39)	*t*(555.8)=−14.25, *p*<.001
Prosocial behaviour (R)*	7.6 (2.27)	5.82 (2.53)	*t*(708) = 8.75, *p*<.001
Impact	1.78 (2.57)	5.71 (2.87)	*t*(525.8)=−18.49, *p*<.001

Note. Degrees of freedom have been adjusted based on results of Levene’s test.*Prosocial behaviour subscale is reverse scored.

SDQ total and subscale scores were then compared between males with DMD and the age and sex-matched normative population data from the MHCYP 2017 cohort (Table 2). The DMD group demonstrated significantly higher mean scores across several SDQ subscales, particularly emotional symptoms, peer problems, and total impact scores (all *ps* < 0.001). The DMD group scored significantly lower in prosocial behaviour (*p* <.001) relative to population norms, suggesting difficulties in social engagement. Detailed *Z* scores and *p* values are presented in Table 3.

**Table 3:** Comparison of data from participants with DMD from the BIND cohort and normative population data from the UK National survey 2017

DAWBA	DMD Group (BIND)	UK National Survey (MHCYP 2017)	Statistical comparison (Z, *p* value)
	(*n* = 238; Rate(%), 95%*CI*)	(*n* = 3,851 Rate(%), 95%*CI*)	
Any disorder	21.01 [16.2–25.9]	12.65 [11.4–13.9]	*Z* = 3.1, *p*<.001
ADHD	8.4 [4.9–11.9]	2.62 [2.1–3.1]	*Z* = 3.18, *p*<.001
ASD	6.72 [3.5–10]	1.92 [1.5–2.4]	*Z* = 2.93, *p*<.001
Anxiety-type disorders	7.98 [4.6–11.4]	0.8 [0.5–1.1]	*Z* = 4.07, *p*<.001
ODD	2.1 [0.4–3.8]	3.6 [3–4.2.2]	*Z*=−1.53, *p*=.12
**SDQ**	**(*n*** **= 238;** ***M***,*** 95%CI*)**	**(*n*** **= 3**,**042;** ***M***,*** CI*)**	
Total score	10.76 [9.4–11.6]	8.7 [8.4–9.0.4.0]	*Z* = 4.48, *p*<.001
Emotional symptoms	2.58 [2.3–2.9]	2.0 [1.9–2.1]	*Z* = 3.59, *p*<.001
Conduct problems	1.84 [1.6–2.1]	1.5 [1.5–1.6]	*Z* = 2.61, *p=*.009
Hyperactivity problems	3.96 [3.6–4.3]	3.6 [3.5–3.7]	*Z* = 2.23, *p*=.025
Peer problems	2.38 [2.1–2.6]	1.53 [1.5–1.6]	*Z* = 6.53, *p*<.001
Prosocial behaviour	7.6 [7.3–7.9]	8.41 [8.3–8.5]	*Z*=−5.02, *p*<.001
Impact	1.78 [1.5–2.1]	0.8 [0.8–0.9]	*Z* = 6.32, *p*<.001

*Note. Due to the individual data not being available for population norms from the national survey*,* comparisons were conducted between group means and CIs using Z tests.*

## BIND cross-site comparison

Lastly, we explored the possible contribution of assessment centre on SDQ scores and DAWBA-derived diagnoses. In a multivariate ANOVA of the SDQ total score there was a significant main effect of study site (*p*s < 0.001) on all subscales, with the exception of SDQ Peer Problems scores (*p*=.799; Supplementary Appendix 5). Bonferroni-corrected post hoc analyses revealed this effect was due to significantly fewer problems reported by parents from the Italian site only. Italian parents rated their children as having relatively fewer emotional problems, conduct problems, overall impact, as well as higher levels of prosocial behaviour.

Intra-group differences were also found for DAWBA-based diagnoses of neurobehavioural disorders (*X*^*2*^(5,238) = 26.26, *p*<.001). Italy (5.6%) and France (16%) had the lowest prevalence, significantly lower than the Netherlands (44%), Spain (25%), and the two UK sites (30%, 40%).

## Discussion

The present study provides a comprehensive evaluation of the neurodevelopmental and psychiatric comorbidities in children and adolescent males with DMD, utilising a standardised and validated online DSM-5 interview (DAWBA) in a prospective, multicentre design. Our findings underscore the significant neuropsychiatric burden associated with DMD and highlight the need for integrated care approaches that address both the physical and psychological dimensions of this condition.

Over 20% of children and adolescent males with DMD met criteria for at least one neurodevelopmental or psychiatric diagnosis, with ADHD (8.4%), ASD (6.7%), and anxiety-type disorders (7.9%) being the most frequent. These rates are broadly consistent with prior studies, though often lower than estimates from smaller or registry-based cohorts, which have reported ADHD prevalence up to 50%, ASD up to 21%, and anxiety disorders up to 60% [3, 19, 40]. Notably, OCD and depression were not identified in this paediatric cohort, which contrasts with existing literature [23, 40, 41]. This discrepancy may reflect age-related onset patterns for these disorders, methodological differences, or limitations in reporting internalising symptoms through parental report.

Genotype–phenotype analyses revealed meaningful associations. ASD was significantly more prevalent in individuals with Dp140- variants, supporting prior evidence that loss of this isoform contributes to neurodevelopmental risk [1, 18]. In contrast, ADHD and anxiety-type disorders were distributed across genotypic groups, suggesting a multifactorial aetiology. Interestingly, oppositional behaviours (ODD) were observed only in the Dp140 + group, potentially reflecting behavioural responses in individuals with milder CNS involvement. Similar genotype-phenotype associations have been reported in other genetic syndromes, such as 22q11.2 deletion syndrome and Fragile X [32, 33, 42], where specific mutations correlate with psychiatric risk. In DMD, CNS involvement may also interact with physical severity, warranting further exploration of links between motor function, cognitive impairment, and emotional wellbeing [14, 43].

Our study highlights the utility of routine neurobehavioural assessments in DMD and demonstrates age-related trends, where mental health and neurobehavioural difficulties are increasingly recognised as a significant contributor to disease burden. Brain comorbidities such as anxiety, depression and ADHD have been linked to reduced quality of life [19], poor treatment adherence [5], and reduced life expectancy in adults with DMD [6]. Additionally, a subset of participants with multiple co-occurring diagnoses was identified, particularly ADHD and ASD. This is clinically relevant, as the presence of complex comorbidities often results in greater care needs, greater functional impairment, and increased caregiver burden [44, 45]. Despite these risks, current care guidelines provide only basic recommendations for psychosocial screening [28], lacking specific protocols for identification and clinical management. In this context, the routine use of brief screening instruments can serve as an initial step in identifying individuals at elevated neurobehavioural risk who may require more comprehensive assessment, including the recently published consortium tool, the BIND Screener, among others [46, 47]. When these screens are positive, a more structured tool, such as the DAWBA, should be incorporated into routine care to support early identification, genotype‑informed stratification, and targeted intervention. This approach is consistent with a stepped‑care framework, in which initial screening helps prioritise referrals for detailed diagnostic evaluation, including clinician‑rated assessments. It also aligns with recent calls for harmonised psychological assessment frameworks in DMD [29].

Present study contributes to existing literature by contextualising neurobehavioural prevalence in DMD relative to children with genomic intellectual disability (IMAGINE-ID) and UK population norms. Compared to IMAGINE-ID, the DMD cohort showed fewer neurodevelopmental diagnoses, potentially reflecting differences in intellectual disability severity and genomic disruption between cohorts. In contrast, relative to population norms, children and adolescent males with DMD exhibited elevated emotional distress, peer relationship problems, and greater functional impact, consistent with prior reports of psychosocial burden in DMD [19, 48]. Notably, hyperactivity scores did not differ, possibly due to reduced mobility—a pattern also observed in mouse models [18]. By employing a comparative framework across three cohorts and using standardised diagnostic tools, this study reinforces the need for genotype-informed, holistic, and lifespan-oriented care models that integrate both neuromuscular and mental health support [6, 28, 29].

Several limitations should be acknowledged. There is a lack of detailed clinical measures of physical health decline, which restricts our ability to fully explore the potential contribution of physical deterioration to emotional difficulties which has been observed in adult DMD [48, 49]. Additionally, reliance on parent-report may underestimate internalising symptoms. Future studies should incorporate direct child interviews, teacher reports, and in-clinic observations. While our genotype-based analyses offer insight into isoform-specific risk, the underlying neural mechanisms remain to be elucidated. Animal models suggest altered glutamatergic and GABAergic signalling, disrupted synaptic scaffolding, and changes to white matter integrity [12, 14, 48, 50]. Combining neuroimaging, genotype, and behavioural data could clarify causal pathways. Lastly, there was a significant cross-site variation observed in both DAWBA-based diagnoses and SDQ scores. These differences may reflect cultural variation in the perception and reporting of mental health concerns, as documented in other multinational paediatric studies [51, 52]. Factors such as health system structure, psychological service availability, and caregiver expectations likely influence how behaviours are interpreted and reported. While site-level variation may have influenced findings, all assessments were centrally rated by a single clinical team to ensure consistency across sites. Using country-specific comparison groups in future research may address some of these concerns.

This study demonstrates the feasibility of standardised neurobehavioural assessment in DMD across a large, multicentre cohort. By integrating genotype data and comparative analyses with both normative and genomic intellectual disability groups, it advances understanding of DMD’s neuropsychiatric profile and its clinical relevance. The findings support the use of tools like the DAWBA for efficient identification of mental health needs in this population. As life expectancy in DMD continues to improve, ensuring that psychological and behavioural wellbeing are embedded within care frameworks will be essential for improving quality of life for affected individuals and their families. 

## Supplementary Information

Below is the link to the electronic supplementary material.

ESM 1 (DOCX.25.3 KB)

## Data Availability

Authors confirm that the datasets supporting the findings of this study are deposited in the Duchenne Data Repository at https://repository.duchennedatafoundation.org/dataset/deep-cognitive-and-behavioral-phenotyping-of-patients-with-dystrophinopaties and under the license CC BY-NC-ND: Attribution-NonCommercial-NoDerivatives 4.0. Interested parties may request access by completing the data access form in the repository’s portal. Access requests will be reviewed in accordance with the repository’s data sharing policies and the terms of the license.
