# Supplementary material for: Understanding the neurobehavioural impact of Duchenne muscular dystrophy: A multicentre European study
Source: Eur Child Adolesc Psychiatry. 2026 Apr 28;35(6):2019–29. doi: 10.1007/s00787-026-02988-7 (PMC13337733; doi:10.1007/s00787-026-02988-7)
Supplement: Supplementary file 1 — (DOCX.25.3 KB) [file 787_2026_2988_MOESM1_ESM.docx]

Supplementary Materials.

**Supplementary Appendix 1. BIND study participating cites**

The study acronym is BIND (for Brain INvolvement in Dystrophinopathies). This study was sponsored by University College London across all sites with identical protocols (Denmark; The Netherlands; France; Spain and Italy), which have been submitted to the local authorities after appropriate translation. In the UK, 2 study sites will take part in the BIND study ([Dubowitz Neuromuscular Centre](https://www.ucl.ac.uk/child-health/research/developmental-neurosciences/molecular-neurosciences/dubowitz-neuromuscular-centre), UCL Great Ormond Street Institute of Child Health and Great Ormond Street Hospital for Children, PI Prof Francesco Muntoni; [John Walton Muscular Dystrophy Research Centre](https://jwmdrc.org/), Newcastle University and Newcastle upon Tyne Hospitals NHS Foundation Trust, PI Prof Volker Straub. The other participating centres include: (a) Denmark: Region Hovestaven DH Hospital, PI Prof John Vissing <http://neuromuscular.dk/>; (b) The Netherlands, Leiden Medical Centre, PI Dr Erik Niks (<https://www.lumc.nl/research/>); and [Stichting  Kempenhaeghe](https://www.kempenhaeghe.nl/), PI Dr Jos Hendriksen; (c) France, [Necker Enfants  Malades  Fondation](https://www.institutimagine.org/en), Pairs, PI: Prof Isabelle Desguerre; (d) Italy, Universita La Cattolica Sacro Cuore, Rome (PI Prof Eugenio Mercuri); and University of Ferrara, Italy (PI Prof Alessandra Ferlini); (e) Spain, [Universidad  Complutense de Madrid](http://www.ucm.es/), PI Dr Ruben Miranda). Additional information on the EU Project website, <https://bindproject.eu/about/>; and on the website of the advocacy groups involved in the project as partners <https://www.worldduchenne.org/project/bind/>. Note that data from the Denmark site is not presented in this manuscript due to differences in ages of recruitment groups (i.e. only adult participants with Becker Muscular Dystrophy were assessed).

Abbreviations of site names are as follows:

University College London (UCL, UK), Newcastle University (UNEW, UK), Kempenhaeghe Centre for Neurological Learning Disabilities (KEM; Netherlands), Universidad Complutense de Madrid (UCM, Spain), Copenhagen Neuromuscular Center, Department of Neurology, Rigshospitalet, Copenhagen (RegionH; Denmark), Imagine Institute des maladies genetiques Necker Enfant maladies foundation Paris (NEM; France) and Universita’ Cattolica del Sacro Cuore, Rome (UCSC; Italy).

**Supplementary Appendix 2.**  Categorisation of SDQ scores for 4–17-year-olds on the 4-band categorisation in parent-completed assessments. Note that scores for Prosocial behaviour are reversed.

|  | Close to average | Slightly raised/lowered | High/low | Very high/very low |
| --- | --- | --- | --- | --- |
| Total difficulties score | 0-13 | 14-16 | 17-19 | 20-40 |
| Emotional problems score | 0-3 | 4 | 5-6 | 7-10 |
| Conduct problems score | 0-2 | 3 | 4-5 | 6-10 |
| Hyperactivity score | 0-5 | 6-7 | 8 | 9-10 |
| Peer problems score | 0-2 | 3 | 4 | 5-10 |
| Prosocial score | 8-10 | 7 | 6 | 0-5 |
| Impact score | 0 | 1 | 2 | 3-10 |

**Supplementary Appendix 3. Parental Wellbeing based in children with or without DAWBA diagnosis.**

A series of independent *t*-tests were performed comparing children who received a DAWBA-based diagnosis relative to those who did not (Diagnosis Present vs. Diagnosis Absent) on the following variables: Family Stress Total Score, Everyday Feelings Questionnaire Parent 1 and respondent’s partner, where available, as well as SDQ Total Difficulties score, see table below for visualisation of means, SDs and sample sizes.

There was a significant difference observed for Family Stress in Diagnosis Present than Diagnosis Absent group (*t*(155)=-5.32, *p*<.001). Further, there was a significant difference in reported parental wellbeing on the EFQ from Parent 1 in Diagnosis Present than the Diagnosis Absent group (*t*(155)=-2.21, *p*=.029). Similar results were seen for reported stress for respondent’s partner (*t*(146)=-2.39, *p*=.022). Lastly, a significant difference in total Total SDQ scores was observed in Diagnosis Present vs. Diagnosis Absent group (*t*(233)=-9.85, *p*<.001), suggesting an overall higher impact in families with children who received a DAWBA-based diagnosis.

A Chi-Squared test was performed on General Health (Very Good, Good, Fair, Bad) vs. DAWBA-based diagnosis (Diagnosis Present vs. Diagnosis Absent). No significant differences were reported between general physical health reported by parents and presence of a DAWBA diagnosis (*X^2^*(3,159)=5.16, *p*=.16).

These preliminary results suggest that while there is no relationship with physical health and neurobehavioural diagnosis in our sample, there is higher parental stress and family wellbeing in children with DMD who screened positive on the DAWBA.

| Measure | DAWBA-diagnosis Present  M, SD (n) | DAWBA-diagnosis Absent M, SD, (n) | Statistical comparison |
| --- | --- | --- | --- |
| Family Stress Total Score | 4.2, 3.39 (30) | 1.39, 2.38 (127) | *t(155)=-5.32, p<.001* |
| EFQ (Parent 1) | 18.53, 5.49 (30) | 15.93, 5.88 (127) | *t(155)=-2.21, p=.029* |
| EFQ (About Respondents Partner) | 18.55, 6.58 (29) | 15.42, 5.15 (119) | *t(146)=-2.39, p=.022* |
| SDQ Total Score | 17.56, 6.23 (50) | 8.82, 5.38 (185) | *t(233)=-9.85, p<.001)* |
| General Health | Very Good=8 Good =9 Fair=12 Bad=2 (31) | Very Good=14 Good =38 Fair=69 Bad=7 (128) | *X^2^(3,159)=5.16, p=.16* |
|  |  |  |  |

**Supplementary Appendix 4.** Participant profiles of those who received more than one diagnosis on the DAWBA.

| Patient | Genotype Group | SDQ_TOTAL_ score | ADHD | Autism | Generalised Anxiety | ODD | Specific Phobia |
| --- | --- | --- | --- | --- | --- | --- | --- |
| 1 | Dp140- | 25 | x | x | x |  |  |
| 2 | Dp140- | 34 | x | x | x |  |  |
| 3 | Dp140Unk | 15 | x |  |  |  | x |
| 4 | Dp140+ | 18 | x | x |  |  |  |
| 5 | Dp140- | 15 | x | x |  |  |  |
| 6 | Dp140- | 32 | x | x |  |  |  |
| 7 | Dp140- | 25 | x | x |  |  |  |
| 8 | Dp140+ | 22 | x |  |  | x |  |
| 9 | Dp140+ | 15 | x | x |  |  |  |

**Supplementary Appendix 5**. Cross-country comparison of SDQ total and subscale scores within the BIND consortium.

| Site | UCL, UK  (*n*=60) | Newcastle, UK  (*n*=20) | Netherlands  (*n*=18) | Spain  (*n*=28) | Italy  (*n*=88) | France  (*n*=23) | Statistical comparison (log transformed) |
| --- | --- | --- | --- | --- | --- | --- | --- |
| SDQ_TOTAL_ | 12.1 (6.9) | 14.6 (7.2) | 12.72 (5.5) | 12.82 (7.3) | **6.71 (5.1)** | 11.57 (5.6) | *F*(5,230)=7.53, *p*<.001 |
| Impact | 2.71 (3.0) | 3.2 (2.8) | 2.5 (2.9) | 2.36 (2.6) | **.44 (1.2)** | 2.13 (2.4) | *F*(5,230)=12.52, *p*<.001 |
| Emotional symptoms | 3.39 (2.7) | 2.85 (2.7) | 2.67 (2.3) | 3.25 (2.7) | **1.76 (2.7)** | 2.7 (2.2) | *F*(5,230)=4.46, *p*<.001 |
| Conduct problems | 2.07 (1.8) | 2.9 (2.2) | 2.33 (2.1) | 2.29 (1.7) | **1.06 (1.2)** | 2.39 (2.3) | *F*(5,230)=6.91, *p*<.001 |
| Hyperactivity problems | 4.2 (2.8) | 5.95 (2.8) | 5.06 (2.4) | 4.57 (2.6) | **2.98 (2.2)** | 3.83 (2.2) | *F*(5,230)=4.9, *p*<.001 |
| Peer problems | 2.44 (1.8) | 2.9 (2.7) | 2.67 (2.1) | 2.71 (2.7) | 1.99 (1.6) | 2.65 (2.0) | *F*(5,230)=4.69, *p=.*799 |
| Prosocial behaviour | 7.19 (2.4) | **5.1 (2.7)** | 6.67 (2.2) | 7.79 (1.9) | 8.7 (1.4) | 6.96 (2.3) | *F*(5,230)=12.38, *p*<.001 |

*Note.* Mean and Standard Deviation scores are included with statistical comparisons conducted on log transformed values due to unequal distribution. Highlighted values showed significant differences in Bonferroni corrected simple effect comparisons.
